# ZC3H15 promotes glioblastoma progression through regulating EGFR stability

**DOI:** 10.1038/s41419-021-04496-9

**Published:** 2022-01-13

**Authors:** Jianbing Hou, Minghao Xu, Hongyu Gu, Dakun Pei, Yudong Liu, Pan Huang, Hongbo Chang, Hongjuan Cui

**Affiliations:** 1 State Key Laboratory of Silkworm Genome Biology, Southwest University, 400716 Chongqing, China; 2 Cancer Center, Reproductive Medicine Center, Medical Research Institute, Southwest University, 400716 Chongqing, China

**Keywords:** CNS cancer, Prognostic markers, Oncogenes

## Abstract

Zinc finger CCCH-type containing 15 (ZC3H15), a highly conserved protein involved in several cellular processes, which was responsible for tumorigenesis and may be a promising marker in myeloid leukemia (AML) and hepatocellular carcinoma (HCC). However, little is known about the biological significance and molecular mechanisms of ZC3H15 in GBM. In this study, we revealed that ZC3H15 was overexpressed in GBM and high ZC3H15 expression was associated with poor survival of patients with GBM. We found that ZC3H15 promoted the proliferation, migration, invasion, and tumorigenesis of GBM cells by activating the EGFR signaling pathway. We also revealed that ZC3H15 reduced EGFR ubiquitination, which was responsible for EGFR protein stabilization. In addition, we demonstrated that ZC3H15 inhibited the transcription of CBL, which was an E3 ubiquitin ligase for EGFR proteasomal degradation. And silencing of CBL could partly abrogate the inhibitory effects on cell proliferation, migration, and invasion of GBM cells induced by ZC3H15 knockdown. Thus, our research revealed the important roles of ZC3H15 in GBM development and provided a brand-new insight for improving the treatment of GBMs.

## Introduction

Glioblastoma which is known as GBM, classified as the WHO IV, possesses the most aggressive ability. The prognosis and survival rate are poor and the median survival time of GBM patients is 14.6 months [1, 2]. Due to its high infiltrative nature, traditional maximum safety resection, radiotherapy and chemotherapy cannot completely remove the intracranial tumor tissue, and eventually lead to recurrence [3]. Two to three people in 100,000 in Europe and North America developed GBM, and of those affected, 90% died within three years [4]. Hence, it is great urgent to find out new treatments for effective GBM therapy.

Zinc finger CCCH-type containing 15 (ZC3H15) also known as DFRP1, a highly conserved gene located at 2q32.1 [5, 6]. ZC3H15 was demonstrated to ortholog with immediate early response erythropoietin 4 (LEREPO4) in mice and induced by erythropoietin [7]. Both ZC3H15 (DFRP1) and DFRP2 possess the DRG family regulatory protein domain, which is pivotal in the interaction of DRG1 and DRG2 [7]. This indicates that ZC3H15 may involve in signaling transduction. Gianni et al. demonstrated that ZC3H15 regulated the NF-κB signaling pathway by interacting with TRAF-2 [8]. Moreover, ZC3H15 also contains the tandem repeat CCCH zinc finger (TZF) domain, which indicates that it may possess the ability to transcriptional regulation and RNA metabolism [9, 10]. At present, there are few studies on ZC3H15, however, the functions of ZC3H15 protein were relatively rich, which have yet to discover and explore.

Casitas B-lineage (CBL) is commonly expressed in cells and belongs to the RING family, contains the Ring finger domain and tyrosine kinase-binding domain in N-terminal. It has been shown that CBL possesses both signaling transduction activity and E3 ubiquitin ligase activity [10, 11]. Indeed, recent studies reported that CBL enhanced the activity of immune cells by reducing PD-L1 expression via the CBL-STAT-AKT-ERK signaling pathway [12]. And CBL played as an E3 ubiquitin ligase to inhibit receptor tyrosine kinase RTKs signaling pathway to further suppress tumor proliferation [13]. Interestingly, CBL protein acts as a tumor suppressor in most cancers, such as non-small cell lung cancer, GBM, and gastric cancer. However, CBL promotes tumorigenesis in resectable pancreatic ductal adenocarcinoma [12, 14, 15]. Due to the multi-function of CBL, it is worth further studying the detailed mechanisms.

The metastasis and proliferation of numerous cancers are driven by epidermal growth factor receptor (EGFR), which is a member of the ErbB family of receptor tyrosine kinases (RTK). Amplification of the EGFR gene and frequent mutations in the EGFR tyrosine kinase domain have recently been demonstrated in cancer patients [16]. Due to the central role of EGFR in cellular progress, the EGFR signaling pathway has become one of the most well-studied signaling pathways in human tumors [17]. As a cross-membrane protein, EGFR is involved in cell to cell communication, cell fate determination, migration, proliferation, and invasion [18–20]. It can also be the substrate for enzymes or the upstream of the signaling pathways. For instance, EGFR promotes tumorigenesis through the EGFR-PI3K/AKT-mTOR-HIF1α signaling pathway. While HIF1α/HIF2α also interacts with EGFR to form a positive feedback [21]. Moreover, in our previous study, the E3 ubiquitin ligase CHIP, degraded EGFR protein which could be aborted by an onco-protein CSN6 [22]. EGFR activation also combines with RTKs heterodimerization or homodimerization and further leads to the activation of various signaling pathways including the Ras-ERK and PI3K-AKT signaling pathways [23–25]. Small molecular inhibitor erlotinib has been discovered recently, it suppresses tumor growth by inhibiting the activation of EGFR. However, inhibiting EGFR in tumors in vivo by erlotinib may not efficient [26–28]. This indicated that drug therapy alone cannot cure tumors completely, and combination therapy will become the trend.

In this study, we found that ZC3H15 was overexpressed in GBM and high ZC3H15 expression was associated with poor survival of GBM patients. ZC3H15 promoted the proliferation, migration, invasion, and tumorigenesis of GBM cells by reducing EGFR ubiquitination degradation. In addition, we found that ZC3H15 elevated the protein stability of EGFR by inhibiting CBL transcription. Thus, our research revealed the important roles of ZC3H15 in GBM development and provided a brand-new insight for improving the prognosis for GBM patients.

## Materials and methods

### Cell lines, reagents, and antibodies

Human GBM cell lines (LN-229, U-251 MG, A172, U-118 MG, and U-87 MG) and normal astroglia cells (SVGP12) were obtained from American Type Culture Collection (ATCC, USA), and all tests were negative for mycoplasma. Cells were cultured by DMEM with 10% fetal bovine serum and 1% Penicillin-Streptomycin solution in 37 °C and 5% CO_2_ incubator. The DMEM media, FBS, and antibiotics were obtained from Thermo Fisher Scientific (MA, USA).

Reagents used in this passage including Cycloheximide (CHX) (Merck, America, CAS:66-81-9), proteasome inhibitor (MG-132) (MCE, China, CAS: 133407-82-6), and Erlotinib (MCE, China, CAS: 183319-69-9).

Antibodies used in this passage including anti-ZC3H15 (Novus Biologicals, USA, CAS: NBP1-81312), anti-EGFR (Cell Signaling Technology, USA, CAS: 4267), anti-p-EGFR (Cell Signaling Technology, USA, CAS: 3777), anti-AKT (Cell Signaling Technology, USA, CAS: 4685), anti-p-AKT (Cell Signaling Technology, USA, CAS: 4060), anti-CBL (Proteintech, China, CAS: 25818-1-AP), anti-PARK2 (Proteintech, China, CAS: 14060-1-AP), anti-STUB1 (Proteintech, China, CAS: 55430-1-AP), anti-Tubulin (Proteintech, China, CAS: 11224-1-AP), anti-Flag (Cell Signaling Technology, USA, CAS: 14793), anti-HA (Proteintech, China, CAS: 51064-2-AP), Anti-actin (Cell Signaling Technology, USA, CAS: 3700), anti-Paxillin (Proteintech, China, CAS: 10029-1-Ig), anti-β-catenin (Cell Signaling Technology, USA, CAS: 8480), anti-ZEB1 (Proteintech, China, CAS: 21544-1-AP), anti-Slug (Proteintech, China, CAS: 12129-1-AP), anti-N-cadherin (Proteintech, China, CAS: 22018-1-AP), anti-E-cadherin (Proteintech, China, CAS: 20874-1-AP), and anti-BrdU (Abcam, USA, CAS: ab6326).

### Database analysis and patient tumor samples

The gene expression data of ZC3H15 was downloaded and analyzed from the CGGA database (http://www.cgga.org.cn/), GlioVis database (http://gliovis.bioinfo.cnio.es/), and TCGA database (https://www.cancer.gov/about-nci/organization/ccg/research/structural-genomics/tcga). Cutoff separating was based on the expression level of ZC3H15, Kaplan–Meier analysis was plotted by CGGA, GlioVis, and R2 database (http://hgserver1.amc.nl/cgi-bin/r2/main.cgi).

Tumor samples were obtained from Chaoying Biotechnology Co., Ltd. (Henan, China). Tissue analysis was approved by the Ethics Committee of Southwest University of China. All the patients provided written informed consent to participate.

### Transfection and Infection

shZC3H15, shCBL and control sequences were recombined into pLKO.1-puro plasmid, and the sequences were listed as below:

Scramble: AGCACACTAGAACCATGTGAA

shZC3H15-1: GCAAGAGATGAAGAACTTGAA

shZC3H15-2: CAGATCCCAAGTCTGTAGTAT

shZC3H15-3: CCTAGAATCAACAGGATGTTT

shCBL-1: GACAAGAAGATGGTGGAGAAG

shCBL-2: CCCTCACAATAAACCTCTCTT

shCBL-3: CCAGTGAGTTGGGAGTTATTA

Flag-tagged full length of ZC3H15 and EGFR recombined into the pCDH-CMV-MCS-EF1-Puro plasmid (Youbio, Hunan, China).

For transfection, lipofectamine2000 (Thermo, USA, CAS:11668019) and specific plasmids were added into the 6-well plate. The medium was changed after 6–8 h. Cells were retrieved after 48 h.

For infection, lentivirus was produced by HEK-293 FT cells. After two times of infection, the medium was refreshed and puromycin was added to screen the cells that are infected.

### Western Blot Analysis and Immunoprecipitation

For the western blot assay, cells were retrieved and lysed on ice by RIPA lysis buffer (Beyotime, China). The supernatant was collected after centrifuge 12,000 rpm at 4 °C for 10 min. The protein was boiled with loading buffer at 96 °C, followed by SDS-PAGE and transmembrane (Bio-Rad, USA). Secondary antibody was incubated after finishing the incubation of primary antibody. The membrane was exposed in the gel imaging system (Qinxiang, China)

For the immunoprecipitation assay, cells were retrieved and lysed on ice by IP lysis buffer (Beyotime, China), The supernatant was collected after centrifuge 12,000 rpm at 4 °C for 10 min, and specific antibodies were added. After 12 h combination, Protein A + G Agarose (Beyotime, China) was added and rotary table for 4 h. Agarose was washed 5 times with PBS followed by loading buffer boiling. Then Western Blot assay was performed.

### BrdU and Immunohistochemistry staining (IHC)

For the BrdU assay, 2 × 10^4^ cells were seeded into the 24-well plate. BrdU was added into the well after the cell adheres to the wall and cultured for 4 h, followed by 4% PFA immobilization, 0.2% Triton X-100 punching, 2 M HCl solution uncoiling. BrdU primary antibody incubated at 4 °C overnight followed by Alexa Fluor^®^ 594 secondary antibody incubation. DAPI was stained before microscope examination.

For the IHC staining, the tissue slides were incubated with specific primary antibodies overnight at 4 °C after deparaffinization, rehydration, hydrogen peroxide treatment, citrate buffer for antigen retrieval, and goat serum blocking. Then slides were incubated with horseradish peroxidase-linked secondary antibody. Diaminobenzidine treatment for visualizing followed by hematoxylin counterstain.

### Ubiquitination assay

Flag-tagged ZC3H15 and HA-tagged Ub plasmids were co-transfected into 293 FT cells, the remaining steps are the same with transfection assay and co-immunoprecipitation assay except the MG-132 was added 8 h before cells were retrieved.

### Protein turnover assay

Specific plasmids were transfected into the cells followed by CHX addition at a concentration of 100 μg/ml, cells were harvested in a time gradient and followed by Western Blot analysis.

### ChIP assay and qRT-PCR

Cells were transfected with CBL promoter truncated plasmid and Flag-tagged ZC3H15 plasmid and lysed on the ice with SDS lysis buffer after two days. After centrifugation, the supernatant was obtained and the anti-Flag antibody was added. The remaining steps were followed by protocols in ChIP Assay Kit (Beyotime, China). SYBR qPCR SuperMix Plus was used for qRT-PCR (Novoprotein, China). The primer pairs are shown in Table 1.

**Table 1:** Primer pairs for real-time PCR and ChIP assays.

*Primer pairs for real-time PCR*	
ZC3H15-F	AACAAAATCCACGTCAGGTAGC
ZC3H15-R	TGCACATACTACAGACTTGGGA
EGFR-F	AGGCACGAGTAACAAGCTCAC
EGFR-R	ATGAGGACATAACCAGCCACC
CBL-F	TAGGCGAAACCTAACCAAACTG
CBL-R	AGAGTCCACTTGGAAAGATTCCT
PARK2-F	GTGTTTGTCAGGTTCAACTCCA
PARK2-R	GAAAATCACACGCAACTGGTC
STUB1-F	AGCAGGGCAATCGTCTGTTC
STUB1-R	CAAGGCCCGGTTGGTGTAATA
*Primer pairs for ChIP assays*	
CBL-2589/-2440-F	CTGTACTCCAGCCTGGGTGAC
CBL-2589/-2440-R	GGCTGAGCGACAAGAGTGAGA
CBL-2279/-2005-F	GGCCAAGGATGGTCTCGAT
CBL-2279/-2005-R	CCTTATTCCATCTGCCTGAAGTAT
CBL-1821/-1559-F	GGTGGCGGGCACTTGTAA
CBL-1821/-1559-R	CCCATTGCCTCGGGAATAA
CBL-1640/-1519-F	GTACCAGGGAGTAGTATAAAACTACAAGG
CBL-1640/-1519-R	GTATAGTTCAGACAGCATCGCAAAA
CBL-1150/-949-F	GAAGGCGGAACTGATACTGACA
CBL-1150/-949-R	GCCCAGGGAACAGAAATGC
CBL-739/-565-F	GTCAAAGGGCCTGGAAGAAGA
CBL-739/-565-R	GGGAGGATGGTGGGAGAAAA
CBL-667/-378-F	GGGCCGCAGCATTCATATT
CBL-667/-378-R	CGCAGTAGCCAGAAACCAAGA
CBL 79/318-F	TCCTTCACGCCCTGCTTCT
CBL 79/318-R	GCACTTCTCCACCATCTTCTTGT

Total mRNA of cells was extracted by the TRIzol method. mRNA was reverse transcripted into cDNA by 1st Strand cDNA Synthesis SuperMix for qPCR (YEASEN, China). And the cDNA was used to perform qRT-PCR.

### MTT Assay

1 × 10^3^ cells were seeded into the 96-well plate. MTT was added and incubated for 2 h and then removed the medium, added 200 μL DMSO. 560 nm absorbency was measured by microplate reader. Repeat the above steps for 6 days. Triplicate was conducted in each sample.

### Migration and invasion analysis

Cells were seeded into transwell (Corning, USA) with 100 µl serum-free medium, and 500 µl complete medium was added into the lower chamber. After 9 h, cells in the upper chamber were erased and cells in the lower chamber were fixed by 4% paraformaldehyde followed by 0.1% crystal violet staining. The procedure for the invasion assay was similar to that for the migration assay, except that the transwell membranes were pre-coated with Matrigel (R&D Systems, USA) and the cells were incubated for 18 h at 37 °C in a 5% CO_2_ atmosphere. The stained cells were examined in the microscope and counted by ImageJ.

### Soft agar colony formation assay

1.2% Agarose as the lower gel was prepared and 1:1 mixed with 2 × DMEM complete medium followed by addition to 6-well plate. 2 ml 2×DMEM containing 1 × 10^3^ cells complete medium was 1:1 mixed with 0.6% Agarose which was the lower gel and quickly added to the culture plate after the solidification of lower gel. After the solidification of the upper gel, the 6-well plate was cultured in the incubator for 2–3 weeks. 0.1% crystal violet was used for staining and examined in the microscope. The colony was counted by ImageJ.

### Dual-luciferase reporter gene assay

The CBL promoter was recombined into pGL3-basic plasmid and co-transfected with indicated plasmids and pGL3-TK plasmid into 293FT cells. The empty vector was considered as the negative control. After 2 days, cells were treated and analyzed by protocol from dual-luciferase reporter assay kit (Promega, USA).

### Gene set enrichment analysis (GSEA)

To determine whether ZC3H15 expression was correlated with the EGFR signaling pathway in GBM, GSEA (version 4.0.3) was used. The CGGA database was downloaded from the Chinese Glioma Genome Atlas (http://www.cgga.org.cn/). The gene sets were obtained from the Molecular Signatures Database (MsigDB, http://software.broadinstitute.org/gsea/index.jsp).

### Xenograft assay

The procedure was performed as described previously [22]. Briefly, 4-week-old female NOD/SCID mice (Beijing Animal Research Center) were purchased and housed in the SPF room. 1 × 10^5^ U-87 MG cells stably transfected with Scramble or shZC3H15 were intracranially injected slowly into the brain of each mouse. Mice were divided into two groups, one group (6 mice for each subgroup) was used for H&E staining, the other group (6 mice for each subgroup) was monitored for survival. Isoflurane anesthesia was used to reduce the pain of the mice when the tumors were harvested. Randomization and single blinding were used for measurement, and the tumor volumes were measured using length (a) and width (b) and calculated using the equation: V = ab^2^/2. The data represent the means ± SD. The bodies were stored at −20 °C and then incinerated by Laibite Biotech Inc. (Chongqing, China). All animal studies were approved by the Institutional Animal Care and Use Committee of Southwest University.

### Statistical process

Triplicates were designed and performed in each experiment mentioned above. Statistical parameters including the sample size and the significance analysis are specified in figure legends. Data was analyzed and shown as mean ± SD. Statistics were analyzed by GraphPad Prism 7.0 whereas the image was analyzed by ImageJ. The Two-tailed Student’s *t* test was used to analyze the data from two groups. The data was considered as significance if the *p*-value < 0.05, *P < 0.05, **P < 0.01, ***P < 0.001.

## Result

### ZC3H15 is commonly upregulated in GBM and correlates with poor prognosis

**Fig. 1:**
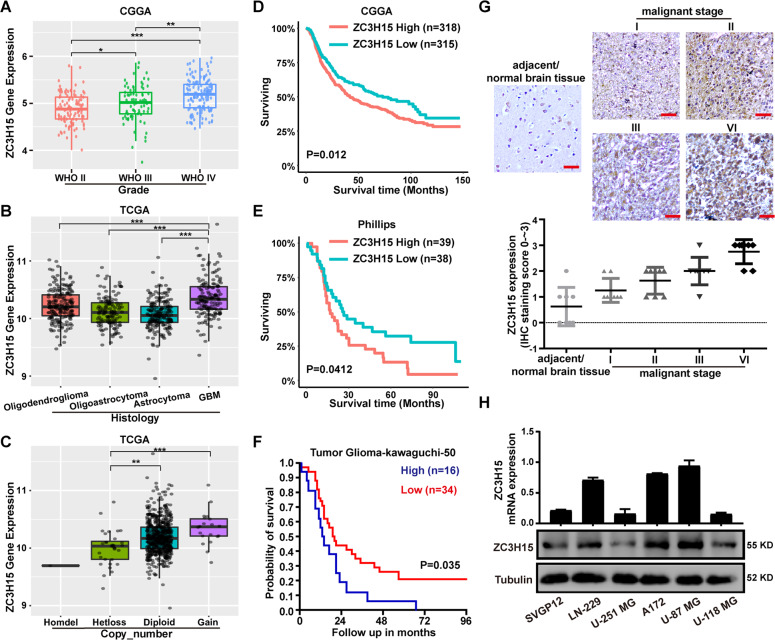
ZC3H15 is commonly upregulated in GBM and correlates with poor prognosis. **A**, **B** ZC3H15 gene expression was obtained from TCGA and CGGA database. Box plot of ZC3H15 expression levels in Grade and Histology glioma set with the log-rank test *P*-values indicated. **C** Box plot of ZC3H15 expression levels in Copy number glioma set with the log-rank test *P*-values indicated. **D**–**F** The correlation between ZC3H15 expression levels and survival rate were obtained from TCGA, Phillips, and Tumor Glioma-kawaguchi-50 and were performed through Kaplan–Meier (K–M) analysis, the log-rank test *P*-value was indicated. **G** Representative immunohistochemistry staining of ZC3H15 expression level in normal tissue and different grades of GBM. And analysis based on the staining of normal tissue (eight samples) and different grades of GBM (eight samples of each group). **H** The protein and mRNA expression profile of ZC3H15 was examined by qRT-PCR and Western Blot assay. All data were expressed as mean ± SD. Student’s *t* test was performed to analyzed significance. *P < 0.05, **P < 0.01, ***P < 0.001.

We downloaded the data from the CGGA database and ranked them by the WHO analysis method, the expression level of ZC3H15 increased with the malignant degree of glioma (Fig. 1A). To further determine the role of ZC3H15 in glioma, we analyzed the characteristics of glioma patients related to ZC3H15 expression based on the CGGA database. As shown in Table 2, ZC3H15 expression was dramatically associated with grade and TCGA subtypes in glioma (Table 2). Isocitrate dehydrogenase (IDH) mutation is commonly observed in glioma, and they are proved to possess the ability to improve the survival rate. We observed that ZC3H15 expression in the IDH mutation group was lower than in the wildtype group (Fig. S1A). Moreover, ZC3H15 expression was also correlated with patients’ age (Fig. S1B). The older the patients, the higher the expression of ZC3H15 in the tumor. We also analyzed the data obtained from the TCGA database, the expression of ZC3H15 is the highest in GBM which is the top level of glioma (Fig. 1B). Furthermore, the clinical data from the TCGA database also indicated that gene copy number gain for ZC3H15 was linked to high expression of ZC3H15 in glioma (Fig. 1C). These results suggested that ZC3H15 played as an onco-protein. To further determine whether high expression of ZC3H15 was linked to the prognosis of tumor patients, survival data from CGGA database was analyzed. As shown in Fig. 1D, high expression of ZC3H15 indicated poor prognosis, same results were also obtained in Phillips and Tumor Glioma-kawaguchi-50 database (Fig. 1E, F). Moreover, IHC staining assays revealed that ZC3H15 expression in malignant gliomas was significantly higher than the adjacent/normal brain tissue, and increases along with malignant stages (Fig. 1G). After that, the protein and mRNA expression of ZC3H15 were examined and demonstrated that higher mRNA level and protein level of ZC3H15 were found in LN-229, A172, U-87 MG cells (Fig. 1H). Taken together, we demonstrated that ZC3H15 was overexpressed in GBM and was associated with a poor prognosis of GBM patients.

**Table 2:** Correlation of ZC3H15 expression with clinicopathological variables in CGGA data sets.

Clinicopathological features		Cases	ZC3H15 expression		*F*	*P*
			Low	High		
Age	≦40	140	84	56	0.271	0.603
	>40	159	67	92		
Gender	Male	180	94	86	0.791	0.375
	Female	121	58	63		
Grade	WHO II	117	66	51	8.335	<0.001
	WHO III	57	38	19		
	WHO IV	124	48	76		
IDH mutation status	Mutation	134	78	56	1.372	0.242
	Wildtype	165	73	92		
1p19q_Codeletion_status	Non-cadel	76	38	38	1.846	0.178
	Cadel	16	5	11		
TCGA_subtypes	Proneural	86	45	41	3.326	0.020
	Mesenchymal	111	59	52		
	Neural	81	33	48		
	Classical	23	15	8		
PRS type	Primary	264	135	129	0.182	0.834
	Secondary	11	4	7		
	Recurrent	23	13	10		

### ZC3H15 is essential for cell proliferation, migration and invasion of GBM cells

**Fig. 2:**
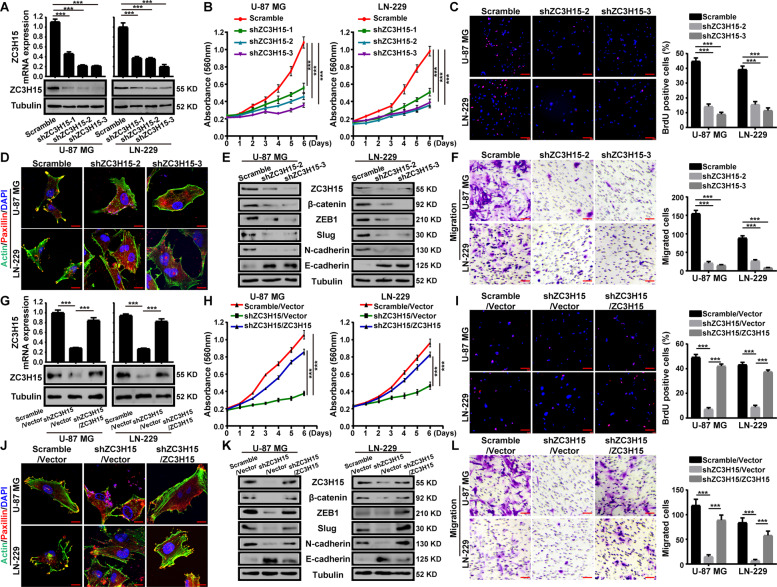
ZC3H15 is essential for cell proliferation, migration and invasion of GBM cells. **A** Western Blot and qRT-PCR assay were performed to prove the knockdown of ZC3H15. **B**, **C** The cells viability and BrdU incorporation were detected by MTT and BrdU assay. BrdU positive cells were counted. **D** ZC3H15-knockdown and control cells were plated on glass slides and stained with anti-Paxillin and anti-actin antibodies. DAPI was used for nuclear staining. **E** The expressions of metastasis-associated proteins were evaluated through western blot analysis in ZC3H15-knockdown and control cells. Tubulin was used as a control for normalization. **F** The effect of ZC3H15 knockdown on cell migration was detected by transwell assay, the migrated cells were counted and analyzed. **G** Western blot and RT-PCR analysis were performed to detect the expression of ZC3H15 in the indicated cells. **H**, **I** The proliferation abilities of indicated cells were analyzed by MTT and BrdU assays. BrdU positive cells were counted and analyzed. **J** Indicated cells were plated on glass slides and stained with anti-Paxillin and anti-actin antibodies. DAPI was used for nuclear staining. **K** The expressions of metastasis-associated proteins were evaluated through western blot analysis in GBM cells. Tubulin was used as a control for normalization. **L** The migration ability of indicated cells was detected by transwell assay. All data were expressed as mean ± SD. Student’s *t* test was performed to analyzed significance. *P < 0.05, **P < 0.01, ***P < 0.001.

According to the expression profile we obtained before (Fig. 1H), U-87 MG and LN-229 cells were utilized in this study. We knocked down the expression of ZC3H15 with three shRNA sequences, shZC3H15 no.1, 2, and 3. The shZC3H15-3 sequence showed more efficiency and was used in the following experiments (Fig. 2A). MTT assays were performed and the data demonstrated that silencing of ZC3H15 significantly inhibited cell proliferation of GBM cells (Fig. 2B). We further examined the bromodeoxyuridine (BrdU) incorporation, and the results indicated that ZC3H15 knockdown significantly reduced DNA synthesis ability (Fig. 2C). Next, flow cytometry analysis was performed to detect the cell cycle and apoptosis in ZC3H15 knockdown and control cells (Figs. S2, S3). We found that silencing of ZC3H15 induced the G1 arrest in GBM cells. However, we did not detect obvious cell apoptosis in the ZC3H15 knockdown group. Moreover, cell senescence experiments were performed and showed that the number of senescent cells increased significantly after ZC3H15 knockdown (Fig. S4). Ectoderm cellular matrix (ECM) remodeling is the main mechanism for GBM invasion and is also the difficulty in curing recurrent brain cancer patients. Paxillin is a cytoskeletal protein involved in actin-membrane attachment to extracellular matrix cell adhesion (adhesion spot) sites [29]. We found that the paxillin protein at the actin-membrane attachment site was dissipated after ZC3H15 knockdown (Fig. 2D). It suggested that ZC3H15 participated in the adhesion and metastasis of tumor cells. After that, we detected the variation of proteins related to cell adhesion. The results demonstrated that the protein levels of β-catenin, ZEB1, Slug, N-cadherin were decreased after ZC3H15 knockdown, while the protein expression of E-cadherin was increased (Fig. 2E). In addition, the abilities of migration and invasion were also reduced in ZC3H15 knockdown GBM cells (Fig. 2F and Fig. S5A).

To further investigate whether ZC3H15 was essential for GBM cells, restoration assay was conducted. Both protein level and mRNA level of ZC3H15 were recovered in ZC3H15 knockdown GBM cells after treatment with ZC3H15 overexpression (Fig. 2G). The abilities of cell proliferation, migration, and invasion of ZC3H15 knockdown cells were also restored in ZC3H15 restored group compared to control cells (Fig. 2H, I, L and Fig. S5B). Meanwhile, the distribution of paxillin and the related protein levels were also restored (Fig. 2J, K). Therefore, these findings indicated that ZC3H15 was involved in cell proliferation, migration, and invasion of GBM cells.

### ZC3H15 activates the EGFR-mediated signaling pathway by increasing EGFR protein stability

**Fig. 3:**
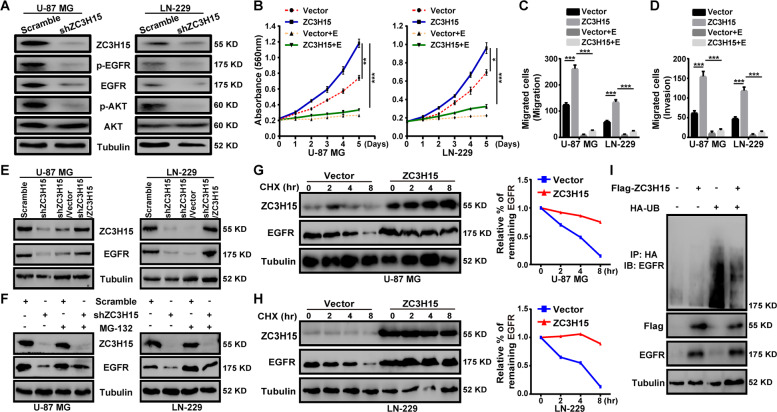
ZC3H15 activates the EGFR-mediated signaling pathway by increasing EGFR protein stability. **A** Western blot analysis was performed to detect the expression of EGFR signaling proteins (EGFR, p-EGFR, AKT, p-AKT) of GBM cells. **B** The inhibitory effect of EGFR inhibitor-erlotinib on the proliferation activity in control or ZC3H15 overexpression cells. **C**, **D** The inhibitory effect of EGFR inhibitor-erlotinib on the invasion and migration in control and ZC3H15 overexpression cells. The number of cells invaded and migrated were counted and analyzed. **E** Western blot analysis was performed to detect the protein expression of EGFR in the indicated cells. **F** Cells were treated with MG-132 for 8 h before harvesting to detect the protein level of EGFR in the indicated cells. **G**, **H** CHX treatment for time gradient (0 h, 2 h, 4 h, 8 h) with the ZC3H15 overexpression and control group. To determine whether ZC3H15 could stabilize EGFR. The gray value was calculated by ImageJ. **I** HA-tagged Ub plasmid and Flag-tagged ZC3H15 plasmid were co-transfected into the cells. The ubiquitinated EGFR proteins were pulled down with anti-HA antibody and immunoblotted with ant-EGFR antibody. All data were expressed as mean ± SD. Student’s *t* test was performed to analyzed significance. *P < 0.05, **P < 0.01, ***P < 0.001.

The protein level of EGFR is highly upregulated in GBM cells, and numerous cellular processes are regulated by EGFR, including cell proliferation, migration, and invasion [30–32]. Interestingly, the GSEA analysis using the CGGA database indicated that high ZC3H15 expression was positively associated with EGFR signaling genes in glioma (Fig. S6). To further validate the data, we next examined the expression and phosphorylation of EGFR and its downstream target protein AKT. The results showed that EGFR and AKT phosphorylation as well as the expression of EGFR were significantly reduced by ZC3H15 knockdown (Fig. 3A). Erlotinib is a small molecular inhibitor that targeted EGFR activation [27, 33]. To determine whether the proliferative effect and the metastatic effect of ZC3H15 on GBM cells were EGFR dependent, we treated ZC3H15-overexpression and control cells with erlotinib. MTT assays demonstrated that erlotinib clearly decreased the promotive effect of ZC3H15 overexpression on cell proliferation (Fig. 3B). Besides, the migration and invasion abilities of GBM cells were also inhibited after treatment with erlotinib (Fig. 3C, D and Fig. S7). Thus, these results demonstrated that ZC3H15 promoted cell proliferation, migration, and invasion by activating EGFR signaling pathway.

We have already shown that the protein level of EGFR was decreased in ZC3H15 knockdown cells, in accordance with expectations, the protein level of EGFR was recovered in ZC3H15-overexpression ZC3H15 knockdown cells (Fig. 3E). In addition, we also detected the mRNA expression of EGFR and found that the mRNA expression of EGFR remained constant (Fig. S8). It has been reported that EGFR may be degraded by the ubiquitin-proteasomal system [34, 35]. Hence, we treated ZC3H15 knockdown cells with the proteasomal inhibitor MG-132. The protein level of EGFR was rescued by treatment with MG-132 (Fig. 3F). Moreover, ZC3H15 overexpression could effectively stabilize EGFR protein from degradation when treating cells with *de novo* protein synthesis inhibitor cycloheximide (CHX) (Fig. 3G, H). To further verify the stabilization ability of ZC3H15 to EGFR, we detected the ubiquitination level of EGFR. ZC3H15 overexpression significantly reduced EGFR ubiquitination (Fig. 3I). Therefore, these results revealed the role of ZC3H15 in activating the EGFR signaling pathway is to reduce the ubiquitination degradation of EGFR.

### EGFR overexpression significantly restored cell proliferation, migration, and invasion of ZC3H15-knockdown GBM cells

**Fig. 4:**
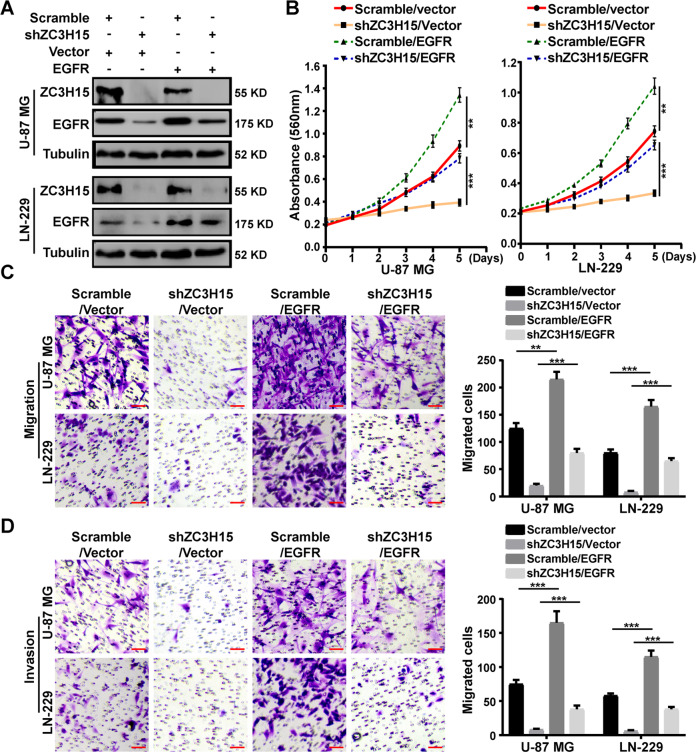
EGFR overexpression significantly restored cell proliferation, migration, and invasion of ZC3H15-knockdown GBM cells. **A** The protein level of ZC3H15 and EGFR were detected in the indicated GBM cells. **B** MTT assays were performed to examine the effect of EGFR overexpression on the cell proliferation of ZC3H15-knockdown GBM cells. **C**, **D** Transwell assays were used to detect the effects of EGFR overexpression on cell migration and invasion of ZC3H15-knockdown GBM cells. All data were expressed as mean ± SD. Student’s *t* test was performed to analyzed significance. *P < 0.05, **P < 0.01, ***P < 0.001.

To confirm whether ZC3H15 regulated GBM progression by targeting EGFR degradation, we overexpressed EGFR in ZC3H15-knockdown U-87 MG and LN-229 cells (Fig. 4A). MTT assays were performed and demonstrated that the inhibition effects of ZC3H15 knockdown on cell proliferation were significantly restored after EGFR overexpression treatment (Fig. 4B). In addition, transwell assays were also performed and indicated that EGFR overexpression dramatically elevated the abilities of migration and invasion of ZC3H15-knockdown GBM cells (Fig. 4C, D). Therefore, these data indicated that ZC3H15 promoted GBM progression by increasing EGFR expression.

### ZC3H15 suppressed the transcription of E3 ligase CBL

**Fig. 5:**
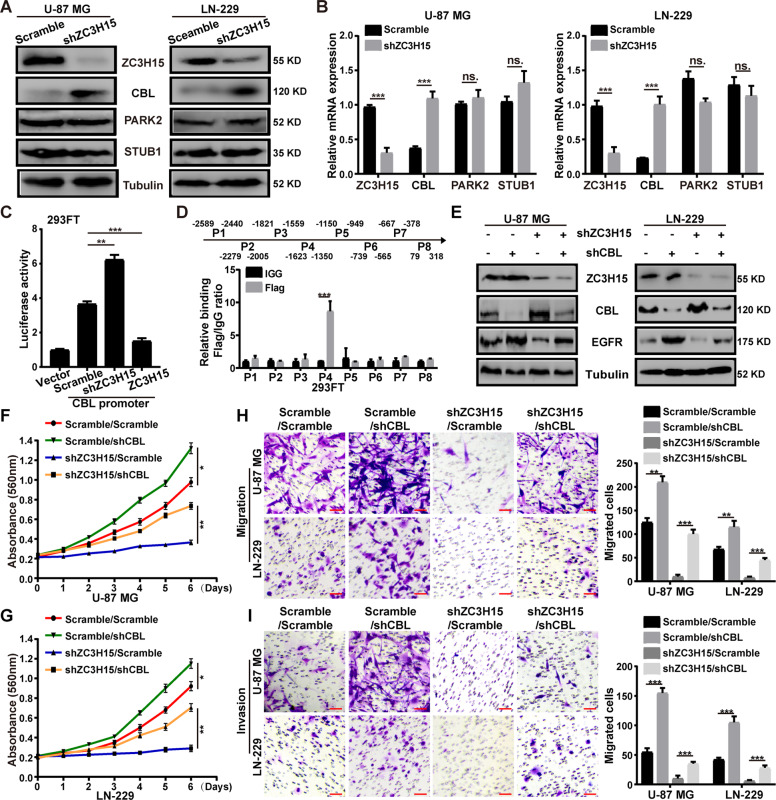
ZC3H15 suppressed the transcription of E3 ligase CBL. **A**, **B** The protein level and mRNA level of several E3 ubiquitin ligases were detected in ZC3H15 knockdown and control cells. **C** The transcriptional activity of CBL in control and ZC3H15 knockdown and overexpression group were detected. **D** ChIP assay was performed by using Flag antibodies. IgG was used as the negative control. **E** The protein levels of ZC3H15, CBL, and EGFR were detected by western blot analysis. **F**, **G** The recovery of cellular proliferation was detected in ZC3H15-knockdown cells followed by CBL knockdown. **H**, **I** The recovery of the migration and invasion abilities in ZC3H15-knockdown cells followed by CBL knockdown. All data were expressed as mean ± SD. Student’s *t* test was performed to analyzed significance. *P < 0.05, **P < 0.01, ***P < 0.001.

It has been reported that EGFR can be ubiquitinated for degradation by several E3 ubiquitin ligases, including CBL, STUB1, PARK2 [22, 36, 37]. Meanwhile, ZC3H15 possesses the ability of transcription regulation [5]. We hypothesis that ZC3H15 may regulate the transcription of some E3 ubiquitin ligases. Hence, we detected the expression of E3 ubiquitin ligases mentioned above in ZC3H15 knockdown cells. Unsurprisingly, the protein level of CBL increased significantly while other E3s remained the same (Fig. 5A). To further verify that ZC3H15 knockdown increases CBL expression through transcriptional regulation, qRT-PCR assay was performed. The mRNA level of CBL increased in ZC3H15-knockdown cells, which indicated that ZC3H15 inhibited CBL transcription (Fig. 5B). The dual-luciferase reporter assay also proved it, increased luciferase activity was observed in ZC3H15-downregulation cells, while the luciferase activity was reduced in ZC3H15-overexpression cells (Fig. 5C). Then, we detected the position of ZC3H15 binding CBL promoter through the ChIP assays, and the results demonstrated that ZC3H15 was enriched at the P4 fragment (Fig. 5D). Our previous data revealed that ZC3H15 decreased CBL level and increased EGFR level (Figs. 3A, 5A). To further verify that ZC3H15 stabilized EGFR through transcription inhibition of CBL, we knocked down CBL expression in ZC3H15-downregulation GBM cells. We designed three CBL-knockdown plasmids, and shCBL-3 showed more efficient and was used in the following experiments (Fig. S9). Then, western blot experiments were performed and demonstrated that the EGFR protein level was dramatically increased after CBL-downregulation treatment (Fig. 5E). Consistently, the abilities of cell proliferation, migration, and invasion of ZC3H15-knockdown cells were significantly increased after CBL-knockdown treatment (Fig. 5F–I). In general, these findings indicated that ZC3H15 inhibited CBL transcription to further stabilize EGFR and promote cell proliferation, migration, and invasion.

### ZC3H15 knockdown inhibits tumor growth and improved prognosis in mice

**Fig. 6:**
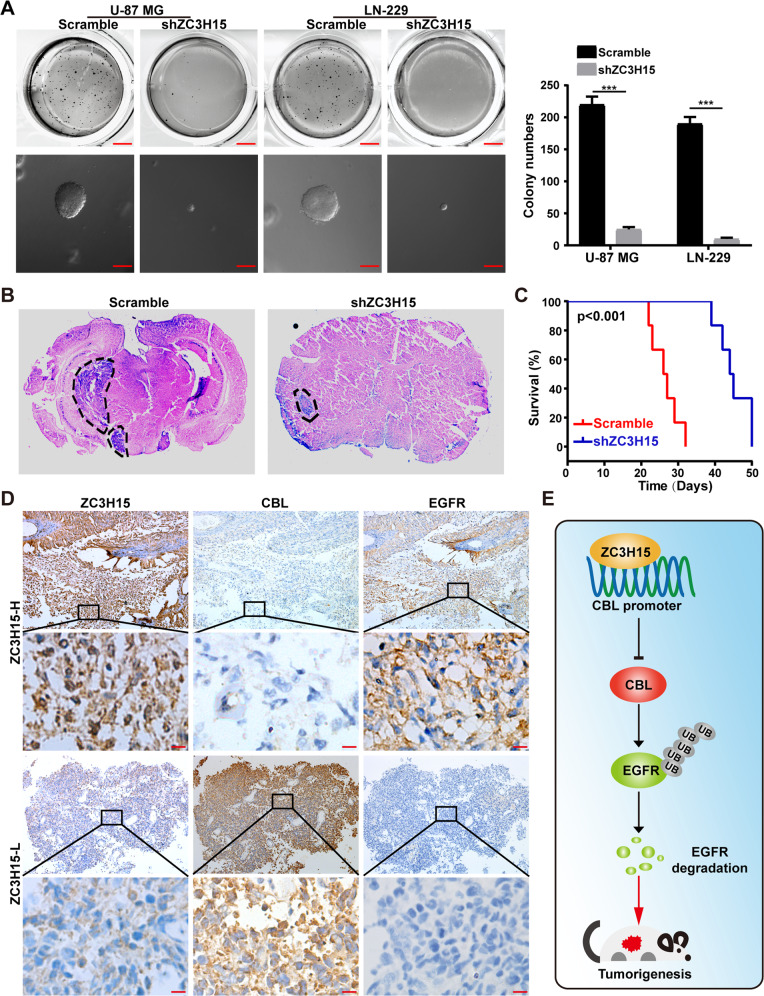
ZC3H15 knockdown inhibits tumor growth and improved prognosis in mice. **A** The colony formation ability of GBM cells was detected by soft agar assay. **B** Orthotopic implantation experiment was performed to assess the in vivo tumor formation capabilities of ZC3H15-knockdown and control cells. **C** The survival curve of mice was plotted based on the living days of mice after the in-situ injection. **D** The expression levels of ZC3H15, CBL, and EGFR were stained and showed in the representative section of tumor samples of GBM patients. ZC3H15-H, ZC3H15 high expression; ZC3H15-L: ZC3H15 low expression. **E** Model of the impact of the ZC3H15 on regulating cell proliferation, migration, invasion, and tumorigenesis of GBM cells. The data were expressed as mean ± SD. Student’s *t* test was performed to analyzed significance. *P < 0.05, **P < 0.01, ***P < 0.001.

To investigate the colony formation effects of ZC3H15 in GBM, soft agar assays were performed and demonstrated that the knockdown group exhibited fewer and smaller colonies (Fig. 6A). To further determine the functional role of ZC3H15 in the tumor growth of GBM cells, the intracranial injection assay was performed. The results showed that ZC3H15 downregulation significantly retarded the tumor formation capabilities of GBM cells (Fig. 6B). Moreover, mice in the ZC3H15-knockdown group survived significantly longer than those in the control group (Fig. 6C). Next, in patient samples, the expression of EGFR was positively correlated to ZC3H15, whereas, CBL was negatively correlated to ZC3H15 and EGFR (Fig. 6D). These results indicated that ZC3H15 promoted tumor growth of GBM cells by targeting the CBL/EGFR signaling pathway (Fig. 6E).

## Discussion

The chemotherapy and radiotherapy have little effects on GBM and surgical removal of the tumor cannot prevent the recurrence [38, 39]. ZC3H15 possesses the ability to promote cell proliferation, migration, invasion, and tumorigenesis. This indicates that overexpression of ZC3H15 in GBM may invade and migrate further in resistance to chemotherapy or radiotherapy. Our previous analysis of ZC3H15 overexpression reduced the survival rate is probably, at least in part proved this. Soft agar can simulate the growth environment in vivo. We found that ZC3H15 downregulation reduced the growth and the colony number of GBM cells, which supports the role of ZC3H15 in tumorigenesis.

EGFR is frequently upregulated in various cancers, including GBM [31]. Betacellulin (BTC) EGF and TGF etc. can bind with EGFR and activate downstream pathway [30]. We have shown that cells with high EGFR expression level were correlated with high proliferation ability [22]. Interestingly, ZC3H15 positively regulated the protein ability of EGFR. Given that EGFR can be ubiquitinated by E3 ligases, meanwhile, we found that the ubiquitination level of EGFR was reduced in the existence of ZC3H15 [40, 41]. It is noteworthy that, ZC3H15 has no Ring finger domain or HECT domain, which is crucial for targeting protein degradation [42]. So, we hypothesis that there is an E3 ubiquitin ligase regulated by ZC3H15 and targeted EGFR for its degradation. Our data added a new pathway for ZC3H15 promoting tumorigenesis which was poorly studied. In addition, small molecular inhibitors of EGFR have been developed, such as erlotinib and gefitinib. These drugs can inhibit the activation of EGFR [43]. However, due to the T790M mutation of EGFR, secondary resistance to these drugs will occur in a year. The future treatment tendency will be genes and drugs combination which is still warranted further study.

Casitas B-lineage (CBL), an E3 ligase which has been studied to involve numerous cellular functions, including immune regulation, proliferation, metastasis, and tumor suppressor [12, 44, 45]. However, disputes existed in the role of CBL played in the tumor, research showed that in non-small cell lung cancer, CBL downregulated PD-L1. So that the tumor can’t confuse T cells. CBL possesses a critical role in the regulation of CD8 (+) T cells and CAR T-cell functions to increase immunity against the tumor. Moreover, CBL is also reported as a tumor suppressor gene in gastric cancer [12, 14, 15, 45]. The detailed mechanisms of CBL in immune regulation remain unclear [12, 45]. Interestingly, ZC3H15 is able to decrease the mRNA and protein level of CBL through transcriptional regulation, thereby promoting tumor growth. It is similar to the way ZC3H15 regulates NF-κB signaling [5].

Our results in animal experiments and tumor samples showed that the promotion effect of the ZC3H15-CBL-EGFR axis to the tumor could be reappeared in vivo. Our Kaplan–Meier analysis from different database is the same as we obtained from mice, which is higher ZC3H15 expression correlates with worse survival time.

In conclusion, we illustrated the role of ZC3H15 as a transcription factor that stabilized the EGFR protein level via inhibiting CBL transcription to promote cancer progression. By regulating this axis, ZC3H15 governed the axis, which was critical in tumorigenesis. Taken together, ZC3H15 was an oncogene acting in tumor cells and with its great functional ability to control numerous cellular progressions. Our study of the ZC3H15-CBL pathway on EGFR expression provided a brand-new insight into tumorigenesis. And these proteins we proposed can be the marker for anti-cancer drugs and combination treatment development.

## Supplementary information

Figure-S1

Figure-S2

Figure-S3

Figure-S4

Figure-S5

Figure-S6

Figure-S7

Figure-S8

Figure-S9

Supplementary metarials

aj-checklist

cddis-author-contribution-form

## Data Availability

All of the data and material in this paper are available when requested.
