# Supplementary figures and images for: ZC3H15 promotes glioblastoma progression through regulating EGFR stability
Source: Cell Death Dis. 2022 Jan 13;13(1):55. doi: 10.1038/s41419-021-04496-9 (PMC8758739; doi:10.1038/s41419-021-04496-9)

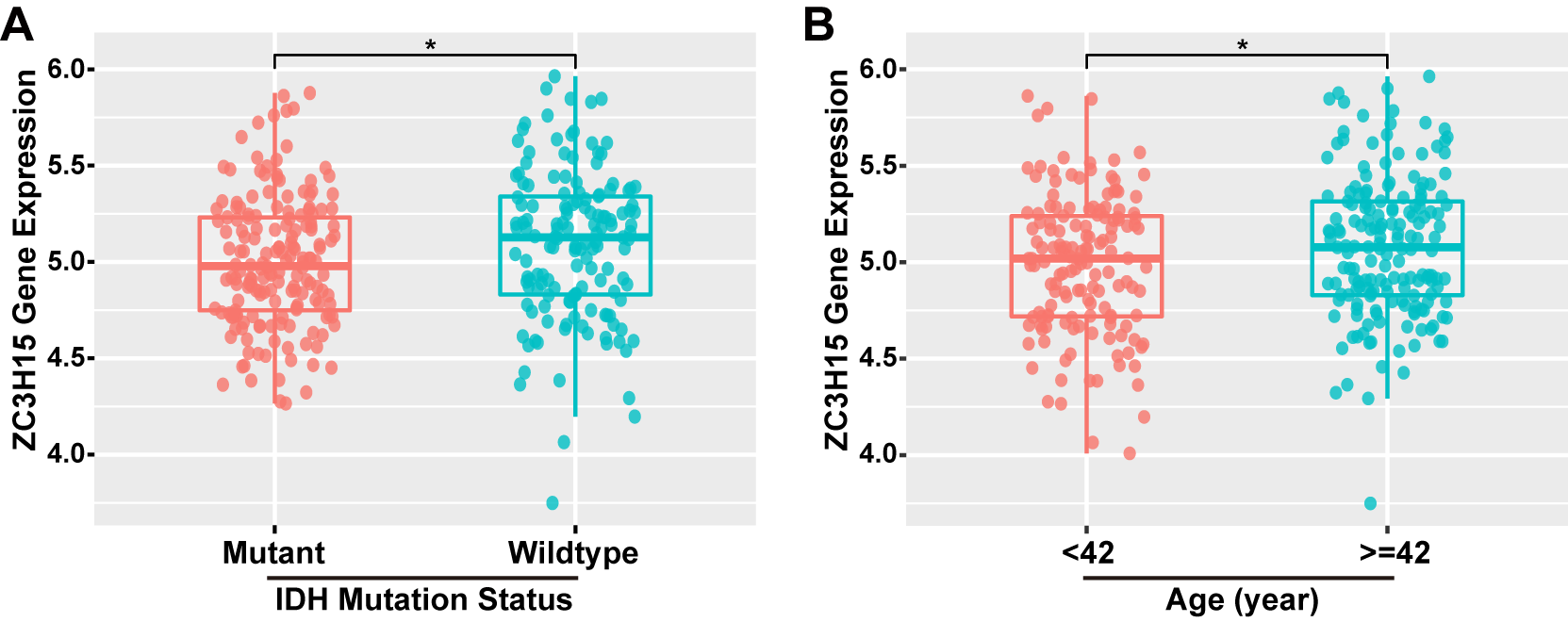

Supplement: Supplementary file 1 — Figure-S1 [file 41419_2021_4496_MOESM1_ESM.tif]

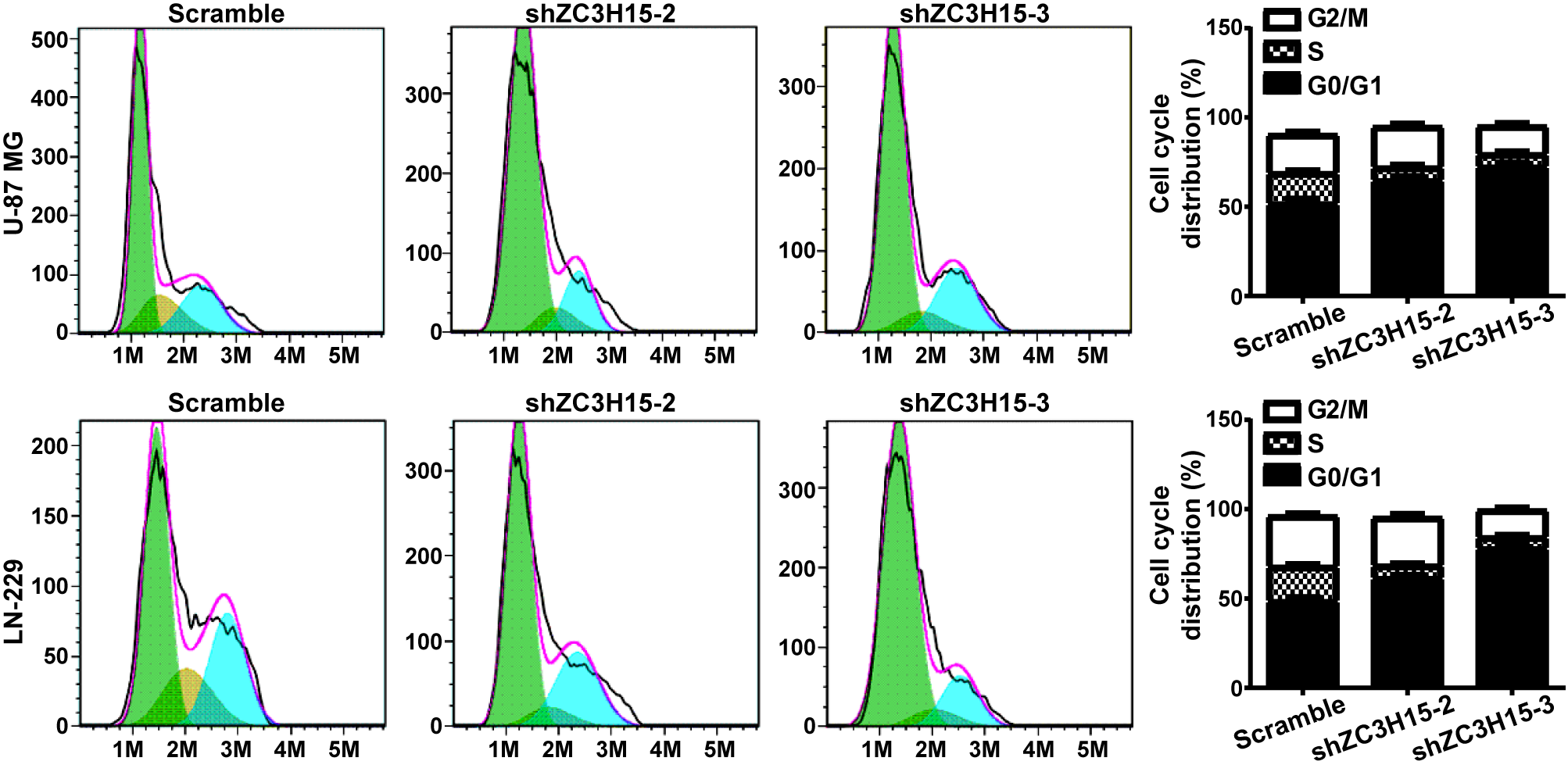

Supplement: Supplementary file 2 — Figure-S2 [file 41419_2021_4496_MOESM2_ESM.tif]

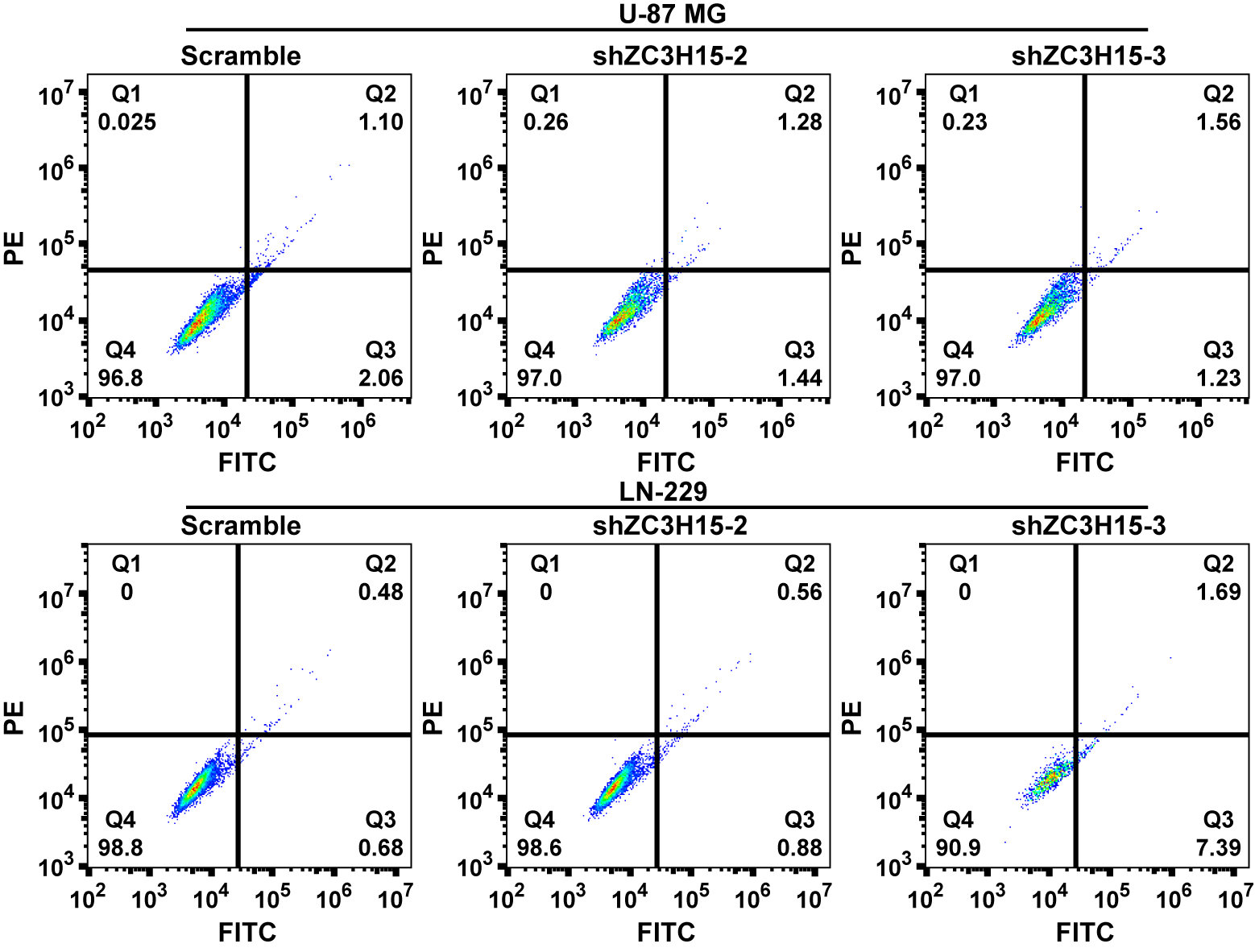

Supplement: Supplementary file 3 — Figure-S3 [file 41419_2021_4496_MOESM3_ESM.tif]

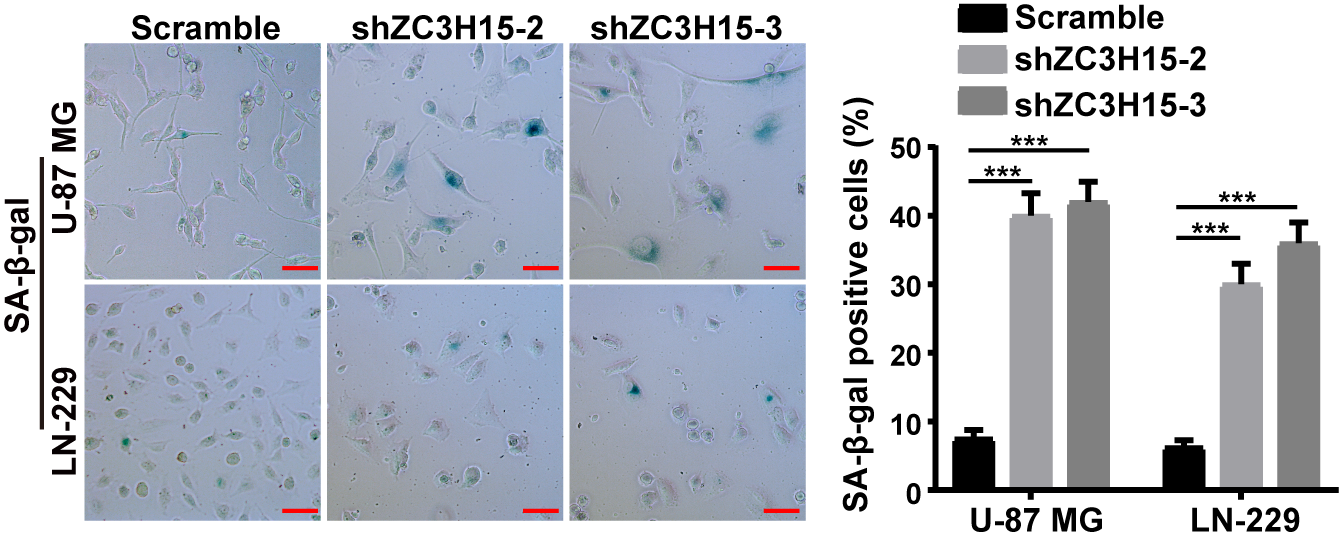

Supplement: Supplementary file 4 — Figure-S4 [file 41419_2021_4496_MOESM4_ESM.tif]

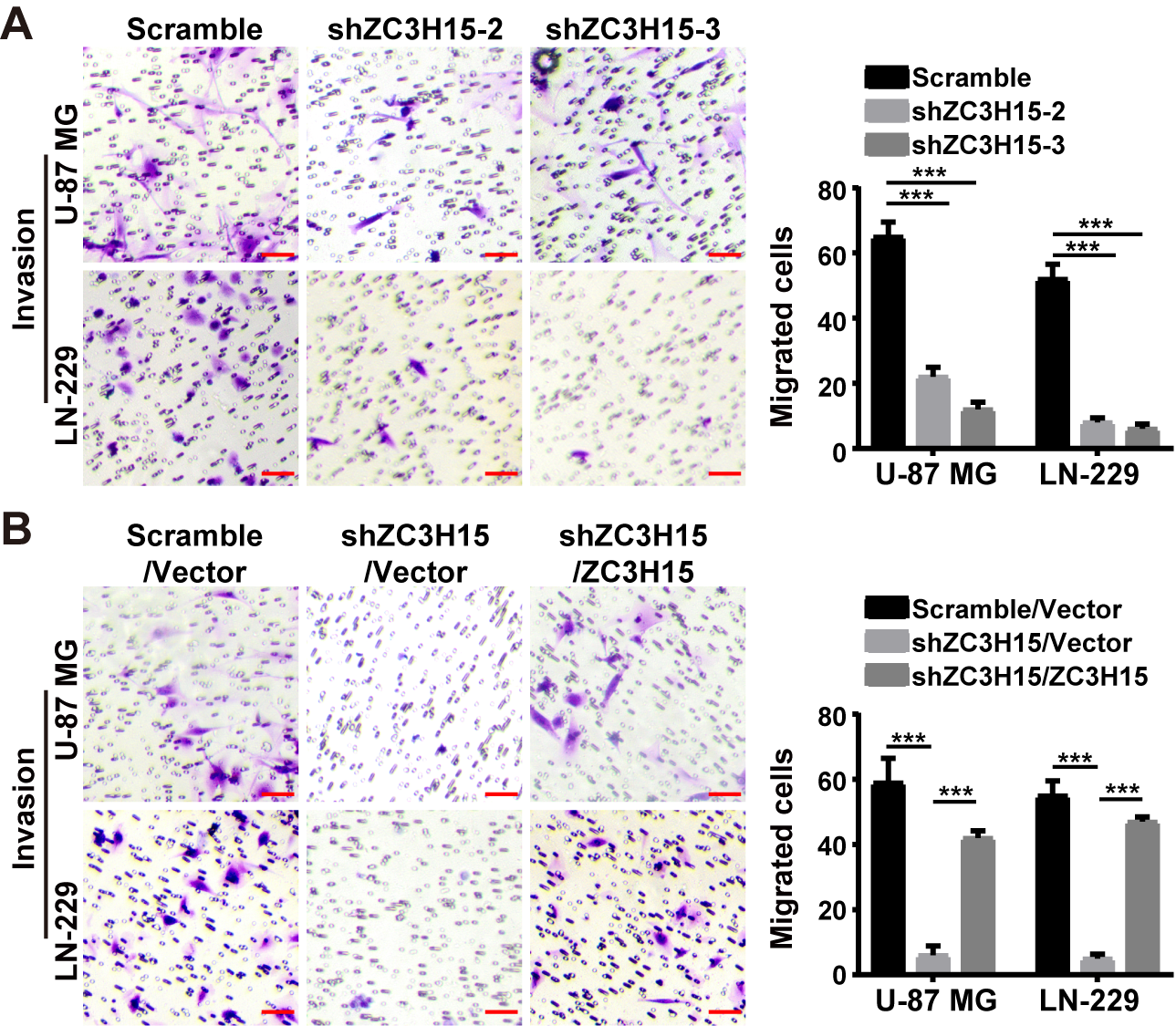

Supplement: Supplementary file 5 — Figure-S5 [file 41419_2021_4496_MOESM5_ESM.tif]

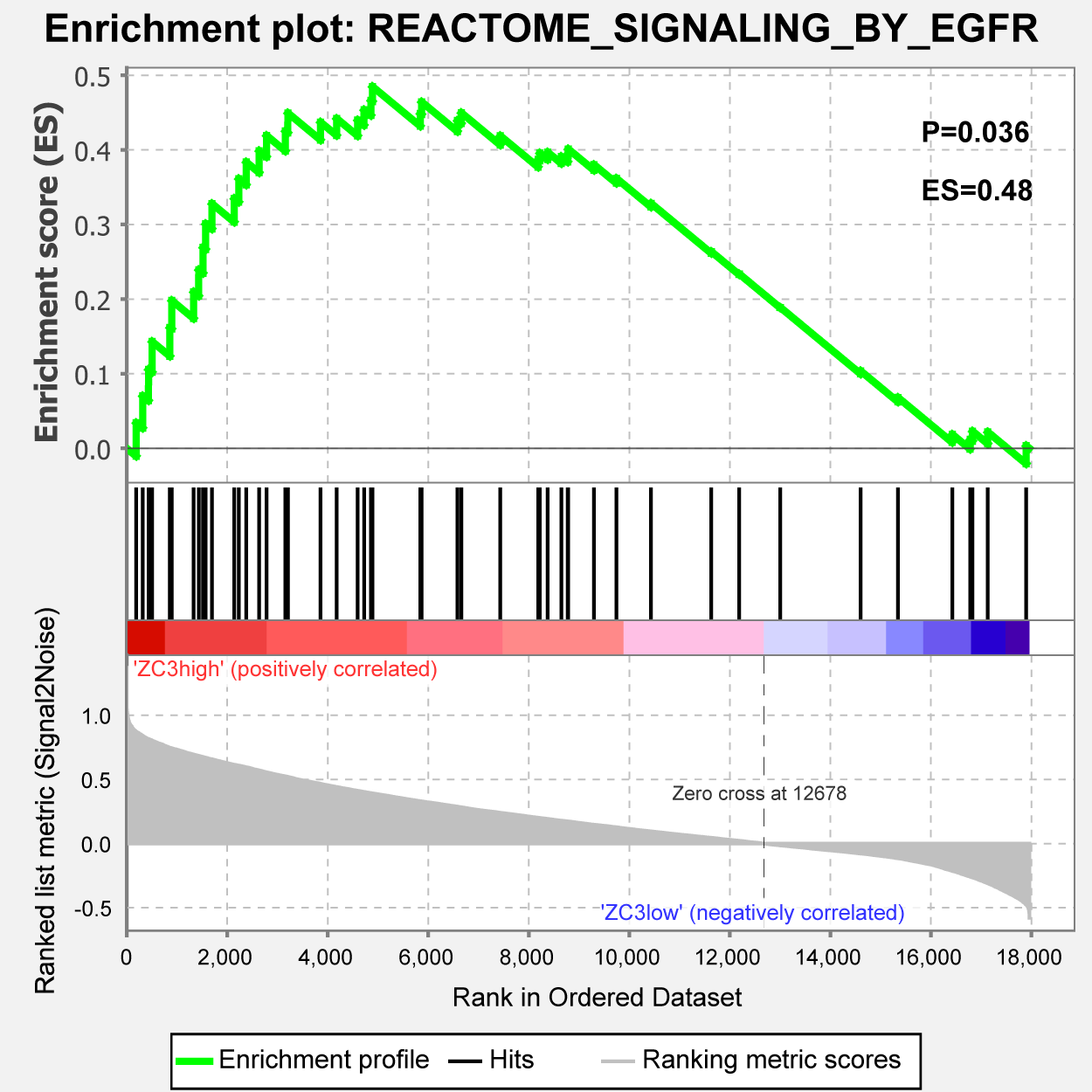

Supplement: Supplementary file 6 — Figure-S6 [file 41419_2021_4496_MOESM6_ESM.tif]

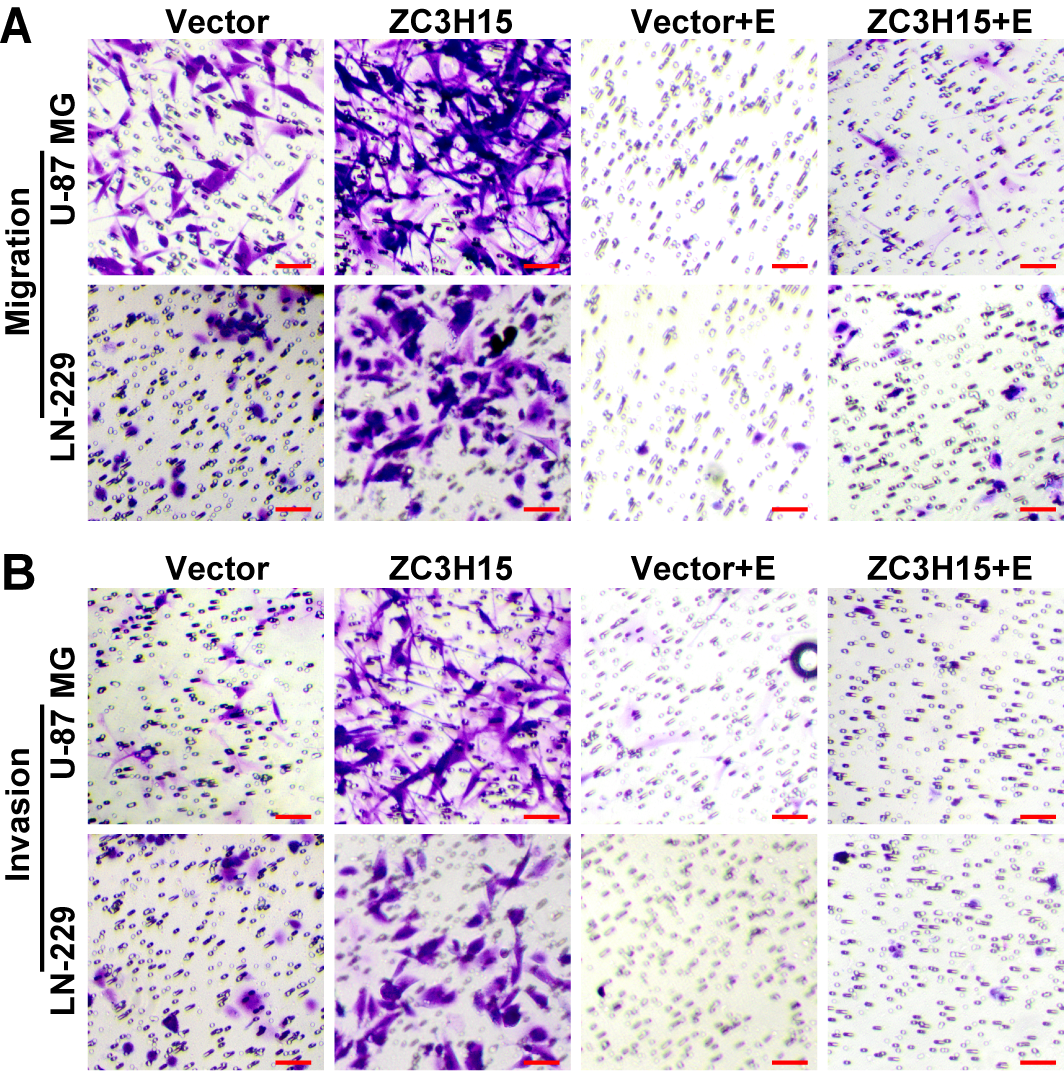

Supplement: Supplementary file 7 — Figure-S7 [file 41419_2021_4496_MOESM7_ESM.tif]

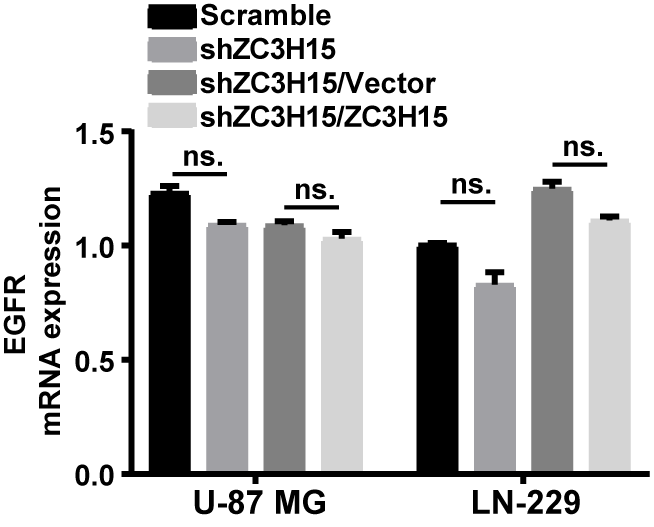

Supplement: Supplementary file 8 — Figure-S8 [file 41419_2021_4496_MOESM8_ESM.tif]

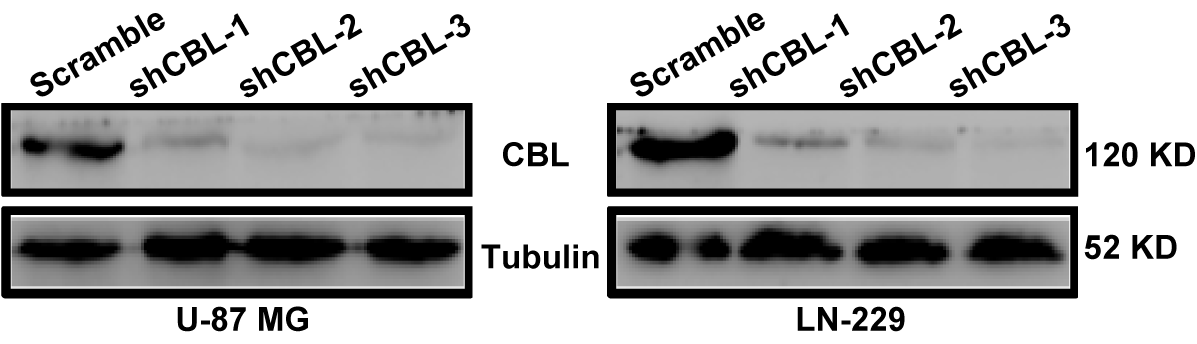

Supplement: Supplementary file 9 — Figure-S9 [file 41419_2021_4496_MOESM9_ESM.tif]
