# Supplementary material for: ZC3H15 promotes glioblastoma progression through regulating EGFR stability
Source: Cell Death Dis. 2022 Jan 13;13(1):55. doi: 10.1038/s41419-021-04496-9 (PMC8758739; doi:10.1038/s41419-021-04496-9)
Supplement: Supplementary file 10 — Supplementary metarials [file 41419_2021_4496_MOESM10_ESM.docx]

**Supplementary Figure 1. (A)** The correlation between ZC3H15 gene expression and IDH mutation status, and P value was indicated. **(B)** The correlation between ZC3H15 gene expression and patient age, and P value was indicated

**Supplementary Figure 2.** Flow cytometry assays were performed to quantify the cell population in each phase of the cell cycle.

**Supplementary Figure 3.** Flow cytometric analysis of cell apoptosis in ZC3H15-downregulation and control cells.

**Supplementary Figure 4.** β-Galactosidase assays were performed to examine the effect of ZC3H15 knockdown on the cell senescence of U-87 MG and LN-229 cells. All data were expressed as mean ± SD. Student’s t-test was performed to analyzed significance. *P<0.05, **P<0.01, ***P<0.001.

**Supplementary Figure 5 (A)** Transwell assays were used to detect the effects of ZC3H15 downregulation on cell invasion of U-87 MG and LN-229 cells. **(B)** Transwell assays were used to detect the effects of ZC3H15 overexpression on cell invasion of ZC3H15-knockdown U-87 MG and LN-229 cells. The number of cells invaded were counted and analyzed. All data were expressed as mean ± SD. Student’s t-test was performed to analyzed significance. *P<0.05, **P<0.01, ***P<0.001.

**Supplementary Figure 6.** The enrichment of ZC3H15 to EGFR pathway was analyzed by GSEA analysis.

**Supplementary Figure 7.** The inhibition effect of EGFR inhibitor-erlotinib on the migration and invasion in control and ZC3H15 overexpression group. The number of cells migrated and invaded were counted and analyzed. All data were expressed as mean ± SD. Student’s t-test was performed to analyzed significance. *P<0.05, **P<0.01, ***P<0.001.

**Supplementary Figure 8.** The mRNA level of EGFR was detected in the indicated cells. All data were expressed as mean ± SD. Student’s t-test was performed to analyzed significance. *P<0.05, **P<0.01, ***P<0.001.

**Supplementary Figure 9.** Western blot assay was performed to prove the knockdown of CBL.
