# Supplementary material for: ZC3H15 promotes glioblastoma progression through regulating EGFR stability
Source: Cell Death Dis. 2022 Jan 13;13(1):55. doi: 10.1038/s41419-021-04496-9 (PMC8758739; doi:10.1038/s41419-021-04496-9)
Supplement: Supplementary file 12 — cddis-author-contribution-form [file 41419_2021_4496_MOESM12_ESM.pdf]

DECLARATION OF CONTRIBUTIONS TO ARTICLE

ADMC

Manuscript Number: CDDIS-21-2490R	Journal Name: *Cell Death & Disease*	(the 'Journal')
Proposed Title of the Contribution: ZC3H15 promotes glioblastoma progression through regulating EGFR stability		(the 'Contribution')
Author(s): Jianbing Hou, Minghao Xu, Hongyu Gu, Dakun Pei, Yudong Liu, Pan Huang, Hongbo Chang, Hongjuan Cui*		(the 'Authors')

For all *CDDis* articles, each person named as an author in the published version must be able to show he or she has contributed substantially to the article.

Authorship credit should be based on 1) substantial contributions to conception and design, acquisition of data, or analysis and interpretation of data; 2) drafting the article or revising it critically for important intellectual content; and 3) final approval of the version to be published. Authors should meet conditions 1, 2 and 3.

Any person who cannot be shown to have made a substantial contribution to the article cannot be listed as an author in the final version. The name of any person who is deemed to have made a minor contribution can, however, appear in the Acknowledgments section of the article.

Please complete the table below to indicate the contributions of all named authors to the manuscript.

Author Full Name:	Specification of Contribution to the Manuscript:
Jianbing Hou	Jianbing Hou designed experiments, obtained and analyzed data, and write the manuscript.
Minghao Xu	Minghao Xu helped design experiments, obtained and analyzed data, and revise the manuscript.
Hongyu Gu	Hongyu Gu helped obtain, analyze some of the data, and revise the manuscript.
Dakun Pei	Dakun Pei helped obtain, analyze some of the data, and revise the manuscript.
Yudong Liu	Yudong Liu helped obtain, analyze some of the data, and revise the manuscript.
Pan Huang	Pan Huang helped obtain, analyze some of the data, and revise the manuscript.
Hongbo Chang	Hongbo Chang designed experiments and revised the manuscript.
Hongjuan Cui	Hongjuan Cui designed experiments and revised the manuscript.

扫描全能王 创建

Please complete the table below to indicate the contributions of all named authors to the figures.

Figure 1:

In Figure 1, JH and MX analyzed the data and prepared panel A-F; HG, DP and YL generated immune-histochemistry data, labelled the image and prepared panel G; PH and HC generated the WB and PCR data and prepared panel H.

Figure 2:

In Figure 2, PH and HC generated the WB and PCR data and prepared panel A, E, G and K;.JH, MX, HG and DP generated MTT, BrdU, IF and transwell data and prepared panel B, C, D, F, H, I, J, L

Figure 3:

In Figure 3, JH, PH and HC generated the WB and PCR data and prepared panel A, E-I; MX, HG, YL and DP generated MTT and transwell data and prepared panel B-D.

Figure 4:

In Figure 4, JH, PH and HC generated the WB data and prepared panel A; MX, HG, YL and DP generated MTT and transwell data and prepared panel B-D.

Figure 5:

In Figure 5, JH, YL and HC generated the WB and PCR data and prepared panel A, B and E; JH and MX generated Luciferase reporter and ChIP data and prepared panel C and D; HG and DP generated MTT and transwell data and prepared panel F-I.

Figure 6:

In Figure 6, PH, YL and HC generated the colony formation data and prepared panel A; JH, MX, HG and DP gerated the xenograft assay and prepared the panel B-D; HC generated and prepared the panel E.

Signed for and on behalf of the Author(s):	Print Name:	Date:
Hongjuan Cui	HONGJUAN CUI	2021.10.10
Jianbing Hou	Jianbing Hou	2021.10.20
HongBo Chong	HongBo Chang	2021.10.20
Dakun Pei	Dakun Pei	2021.10.20
Pan Huang	Pan Huang	2021.10.20

扫描全能王 创建

Signed for and on behalf of Author(s):	Print Name:	Date:
Hongyu Gu	HONGYU GU	2021.10.20
Minghao XU	MINGHAO XU	2021.10.20
Yudong Liu	YUDONG LIU	2021.10.20

扫描全能王 创建
